# Spot Urinary Creatinine Concentration in Patients with Chronic Heart Failure Identifies a Distinct Muscle-Wasting Phenotype with a Strikingly Different Risk of Mortality

**DOI:** 10.3390/biomedicines11092342

**Published:** 2023-08-23

**Authors:** Jolanta Malinowska-Borowska, Marta Buczkowska, Sylwia Duda, Apolonia Stefaniak, Jacek Niedziela, Jolanta Urszula Nowak, Jadwiga Nessler, Karol Adam Kamiński, Mariusz Gąsior, Piotr Rozentryt

**Affiliations:** 1 Department of Chronic Diseases and Civilization-Related Hazards, Faculty of Public Health in Bytom, Medical University of Silesia in Katowice, 41-902 Bytom, Poland; mbuczkowska@sum.edu.pl (M.B.); sduda@sum.edu.pl (S.D.); apolonia.stefaniak@gmail.com (A.S.); jacek.niedziela@gmail.com (J.N.); prozentryt@sum.edu.pl (P.R.); 2 3rd Department of Cardiology, Silesian Centre for Heart Disease, Faculty of Medical Sciences in Zabrze, Medical University of Silesia, 41-800 Zabrze, Poland; nowjola@wp.pl (J.U.N.); m.gasior@sccs.pl (M.G.); 3 Department of Coronary Disease and Heart Failure, Institute of Cardiology, Jagiellonian University Medical College, 31-155 Krakow, Poland; jadwiga.nessler@uj.edu.pl; 4 Department of Population Medicine and Lifestyle Diseases Prevention, Medical University of Bialystok, 15-269 Bialystok, Poland; karol.kaminski@umb.edu.pl

**Keywords:** spot urine creatinine, body composition, fat-free mass, heart failure, phenotype

## Abstract

Background. There is a raising awareness that heart failure (HF) is a highly heterogeneous, multiorgan syndrome with an increasing global prevalence and still poor prognosis. The comorbidities of HF are one of the key reasons for presence of various phenotypes with different clinical profile and outcome. Heterogeneity of skeletal muscles (SMs) quantity and function may have an impact on patient’s phenotype. Aim. We intended to compare clinical characteristics of phenotypes defined by a combination of various SM mass taken as a fat-free compartment from DEXA scans and different levels of SUCR (Spot Urinary Creatinine). All-cause mortality with mortality predicted by MAGGIC in such phenotypes were compared. Methods. In 720 HF patients with reduced ejection fraction (age: 52.3 ± 10 years, female: 14%, NYHA: 2.7 ± 0.7, LVEF: 24.3 ± 7.3%), admitted to the hospital for heart transplantation candidacy assessment, morning SUCR along with body composition scanning (DEXA) was performed. All study participants were dichotomized twice, first by low or normal appendicular muscle mass index (ASMI) and second by SUCR (Spot Urinary Creatinine) < and ≥of 1.34 g/L. Four study groups (phenotypes) were created as combinations of lower or higher SUCR and low or normal ASMI. Results. Low ASMI was found in 242 (33.6%) patients, while the remaining 478 had normal muscle mass. In 446 patients (61.9%), SUCR was <1.34 g/L. During 3 years of follow-up, 223 (31.0%) patients died (all-cause). The phenotype of lower both ASMI and SUCR was associated with the highest mortality. The death rate in phenotype with both low ASMI and SUCR exceeded by 70% the risk estimated by MAGGIC. This difference was significant as judged by the 95% confidence interval for MAGGIC estimation. In Cox regression analysis adjusted for MAGGIC and parameters known to increase risk, the relative risk of patients with phenotype of low both ASMI and SUCR was elevated by 45–55% as compared to patients with all other phenotypes. The protective role of higher SUCR in patients with muscle wasting was, therefore, confirmed in Cox analysis. Conclusions. Measurement of SUCR in HF patients can identify clinical phenotypes with skeletal muscle wasting but strikingly different risk of death that is actually not captured by MAGGIC score. The higher level of SUCR was associated with similar risk independently of presence of muscle wasting. As the analysis of SUCR is cheap and easy to perform, it should be further tested as a potentially useful biomarker, which may precisely phenotype HF patients independently of their skeletal muscle status.

## 1. Introduction

A global epidemic of heart failure (HF) has become a reality. Among the most important reasons for HF outbreak there are population aging, lower births rates in industrialized regions and more efficient treatment of acute cardiovascular diseases. A recent meta-analysis shows that the prevalence of HF may exceed 4% in the some regions [1], which is twice as high as previously thought [2]. It is estimated that approximately 64 million people suffer from HF globally [3,4].

Despite significant progress in therapy and slight improvement of survival, HF is still associated with morbidity and mortality comparable to several cancers [5] with short life expectancy in more advanced stages, severely impaired quality of life and high rates of disability [6,7].

In clinical practice, HF trajectory is highly variable and the use of validated risk scores may help tailor therapy to the individual disease course.

Therefore, prognostic tools and indicators carrying information about HF patients’ risk of death are sought. Some novel biomarkers include vascular stiffness and endothelial function. According to Baran et al., vascular stiffness is associated with outcome after degenerative aortic valve stenosis intervention, but it cannot be used as an independent outcome predictor [8]. Impaired endothelial function of both peripheral vessels and coronary epicardial vessels is also a common characteristic of heart failure and has been associated with worse cardiovascular outcomes [9]. These methods are expensive, difficult to implement and rarely available. Nowadays, the most popular risk predictors are based on clinical characteristics and some biomarkers [10]. However, none of them include muscle wasting, which has been shown as a potent risk factor for mortality [11,12]. As SMs are almost the exclusive source of creatinine, it is believed that in steady-state, urinary excretion rate of creatinine (UCER) may serve as a useful proxy for skeletal muscle mass [13]. In fact, studies showing association of higher mortality in patients with HF with low UCER or more recently also with lower spot urinary creatinine concentration (SUCR) have interpreted these phenomena as SM wasting-related effects [14,15,16]. However, in all aforementioned studies, direct measurements of fat-free mass were not available, making such conclusions speculative. Additionally, a recent study reported only weak correlation between fat-free mass measured by dual X-rays absorptiometry (DEXA) and SUCR levels [17]. Thus, a lot of uncertainty remains on the mechanism linking low UCER and SUCR with mortality. It is possible that factors beyond muscle wasting may play a role in it.

Creatinine excreted into urine is a product of creatine breakdown in muscles [18]. The creatine in its phosphorylated form—phosphocreatine—serves as a key molecule to provide phosphates to ADP and restore cellular pool of ATP [18]. Therefore, creatine availability is of utmost importance for functional integrity of high-energy-consuming organs, such as SM and the heart [18]. In HF patients, either SM or the heart are creatine depleted [19,20]. Thus, it is possible that low UCER and SUCR reflect not only low SM mass, but also impaired muscle function caused by energetic constrains. According to this way of thinking, the various levels of SUCR may reflect metabolic deficiencies and create distinct phenotypes across spectrum of SM mass. 

In our current work, we aimed to analyze clinical characteristics of groups with different combinations of fat-free mass measured by DEXA and various levels of SUCR. Furthermore, we compared all-cause mortality in such groups with mortality predicted by validated prognostic score.

## 2. Materials and Methods

### 2.1. Study Group 

Data collected in the Prospective Registry of Heart Failure of the University Department were used in this study. We selected HFrEF patients, i.e., patients with HF and reduced left ventricle ejection fraction (LVEF) ≤40%, diagnosed according to criteria published by the European Society of Cardiology [2], aged >18 years and with HF duration of more than 6 months, recruited in an outpatient setting from January 2004 to March 2013. All patients were referred as potential candidates for transplantation based on clinical presentation in referral center. After elimination of reversible causes of heart failure, correction of aggravating factors and commencement of optimal therapy some of patients improved to NYHA class II or even I. For all recruited patients, HF could be confirmed with 1-month precision and records concerning body weight before the first diagnosis of HF and minimal weight during HF should be available. 

The onset of HF was defined as a month in which medical records prepared by a cardiologist demonstrated the coexistence of LVEF ≤ 40% with typical signs and/or symptoms of HF. The diagnosis was also supported by the finding of elevated levels of N-terminal pro-brain natriuretic peptide (NTproBNP). The maximum unchanged therapy had to be longer than 1 month before the index date. The pre-HF maximal body weight was defined based on the outpatient medical records as the highest weight within a year, but not later than 2 months before HF diagnosis. If several weights were available for the patient and did not differ by more than 2 kg, the mean value was calculated and taken as pre-HF maximum weight. If the difference was greater than 2 kg, the patient was excluded. The lowest body weight was defined as the minimum body weight when the attending cardiologist did not change diuretics or did not perceive signs and/or symptoms of fluid retention upon clinical examination.

Patients having active infection, liver disease with enzyme levels four times higher than normal, active bleeding, known neoplasm and who had undergone bariatric surgery or surgery reducing intestinal absorptive capacity were excluded. 

Of 1168 registered participants, 720 fulfilled the study criteria. Medical records of this study group were reviewed and comorbidities such as hypertension, diabetes mellitus and hypercholesterolemia were recognized based on clinical history, current medication or actual measurements of the respective variables. History of smoking was defined as current or previous use of tobacco products. 

One spot urine sample was collected per person on the index day. Blood samples were drawn in a standardized manner in the morning, between 8 and 10 am, from patients who had been fasting for at least 8 hours and resting in a supine position in a quiet, environmentally controlled room for 30 min. Blood was immediately centrifuged at 4 °C and stored at −75 °C for further analyses. All procedures were undertaken in accordance with Helsinki Declaration. The protocol was reviewed and accepted by the Ethical Committee of Medical University of Silesia in Katowice (NN-6501-12/I/04). All patients expressed their informed, written consent.

### 2.2. Study Procedures

Body mass and height were measured on the day of blood sampling (index date) using a certified scale (Redwag, Zawiercie, Poland). Body mass index (BMI) was calculated by dividing weight in kilograms by height in meters squared. Only index weight was directly measured. Pre-HF and minimal HF body weights were obtained from medical records as described above. Pre-HF BMI, min HF BMI and index BMI corresponding to maximal pre-HF, minimal HF and index weights were defined in this study. According to our previously described concept we have calculated four indices reflecting edema-free weight trajectory from HF onset until index date, i.e., weight loss, catabolic component, anabolic component and catabolic/anabolic balance: 

1. Weight loss [%] = 100 × (pre-HF BMI − index BMI)/pre-HF BMI;

2. Catabolic component = 100 × (min HF BMI − pre-HF BMI)/pre-HF BMI, (negative value or zero if min HF BMI = pre-HF BMI);

3. Anabolic component = 100 × (index BMI − min HF BMI)/min HF BMI, (positive value or zero if index BMI = min HF BMI);

4. Catabolic/anabolic balance = Catabolic component − anabolic component [21].

Sonos-5000 Hewlett-Packard Ultrasound Scanner (Hewlett-Packard, Andover, MA, USA) was used to measure LVEF from the apical four-chamber view and calculate it with the following formula:

LVEF = [(end-diastolic volume − end-systolic volume)/end-diastolic volume] × 100

Body composition analysis was performed with the use of dual X-ray absorptiometry (DEXA) with a pencil beam Lunar DRX-L device (General Electric, Brussels, Belgium). Compartments of body mass were measured and used in further analyses. Commercially available reagents (Roche Diagnostics, Rotkreuz, Switzerland) allowed to measure hemoglobin, standard indices characterizing erythrocyte hemoglobin, such as MVC, MCH and MCHC, activity of gamma glutamyl transpeptidase (GGTP) and serum and urinary creatinine, serum cystatin C, N-terminal pro-brain natriuretic peptide (NTproBNP) and serum sodium. Kidney function was estimated based on an equation from the Modification of Diet in Renal Disease (MDRD) [22]:

eGFR_MDRD_ = 186 × plasma creatinine [mg/dL]^−1.154^ − age [years]^−0.203^ × 0.742 (if female)

Appendicular skeletal muscle index was calculated according to the equation:

ASMI [kg/m^2^] = ASM [kg]/height^2^ [m^2^]

### 2.3. Statistics 

Study groups were defined based on two variables. First, all patients were assigned to the low or normal ASMI category taking advantage of sex-specific cut-offs (7.0 kg/m^2^ for men and 5.5 kg/m^2^ for women) proposed in the revised consensus document by European Working Group on Sarcopenia in Older People (EWGSOP2) in 2019 [23]. In the second step, receiver operating characteristics were calculated for SUCR and 3-year mortality. All patients were allocated to either low or higher SUCR group based on the SUCR cut-off value that optimally discriminated alive patients from those that had passed away (index Youden). The SUCR value of 1.314 g/L was found to be optimal. The area under the receiver operative curve was 0.563 ± 95%CI: 0.519–0.607, *p* = 0.004. The final groups were constructed as low ASMI and low SUCR (phenotype 1), low ASMI and higher SUCR (phenotype 2), normal ASMI and low SUCR (phenotype 3) and normal ASMI and higher SUCR (phenotype 4), and later, they will be considered as distinct skeletal muscles phenotypes. 

Categorical variables are presented as percentages. Quantitative normally distributed data are presented as mean and standard deviation, whereas non-normally distributed data are presented as median and interquartile ranges (IQR). Variables of study groups were compared using Kruskal–Wallis or Chi-square tests where appropriate. In parameters with differences identified based on Kruskal–Wallis analysis, two kinds of multiply comparisons were performed. First, we compared both phenotypes of muscle wasting (low ASMI) with phenotype of preserved muscle and higher SUCR. Secondly, we compared in pairs phenotypes of muscle wasting, then both phenotypes of preserved muscles. Bonferroni correction between subgroups were performed where appropriate.

The mortality rates in patients with study groups were compared using a Chi-square test, and in the next step, compared with respective 3-year risk estimation calculated based on MAGGIC score [24]. MAGGIC score (Meta-Analysis Global Group in Chronic Heart Failure) is a widely used score to predict all-cause mortality in HF patients and determine the best approach.

We checked whether observed mortality rates have fallen within or outside 95% confidence intervals as computed based on the MAGGIC score. The Kaplan–Meier method served to show and compare (Chi-square test) the probability of survival for patients assigned to study groups at 3 years of follow-up. 

Finally, we used the Cox proportional hazard method to estimate the risk of all-cause mortality at 3-year follow-up in the study groups, taking group 1 with the highest mortality as a reference. The risk was presented as hazard ratio with 95% confidence intervals and showed in crude model and in models adjusted for clinically important confounders. In the final Cox analysis, we used estimated MAGGIC risk score for 3-year mortality for model adjustment. For all analyses, the significance level was set at 0.05 (two-tailed), and all calculations were performed using the software package of Statistica v.13 (Statsoft, Poland).

## 3. Results

### 3.1. Study Cohort Characteristics

There were 720 HF patients with a mean age of 52.3 years who met the study inclusion criteria with no reason for exclusion. Among them, nearly 90% were male and 57% had ischemic etiology of HF. Most patients were in NYHA class II and III and had mean LVEF of about 24%. The complete clinical data, anthropometry, body composition characteristics, laboratory data, medical history and current therapy of patients included in the analysis are shown in Table 1. 

The mean mortality rate as estimated using MAGGIC score was 24.7 (95% confidence intervals: 19.1–34.2%) and it was numerically slightly lower than 31% mortality observed during 3 years of follow-up. Taking into account confidence intervals for risk estimation, this difference was not statistically important (Table 1). 

### 3.2. Appendicular Skeletal Muscles Phenotyping

**Figure 1:**
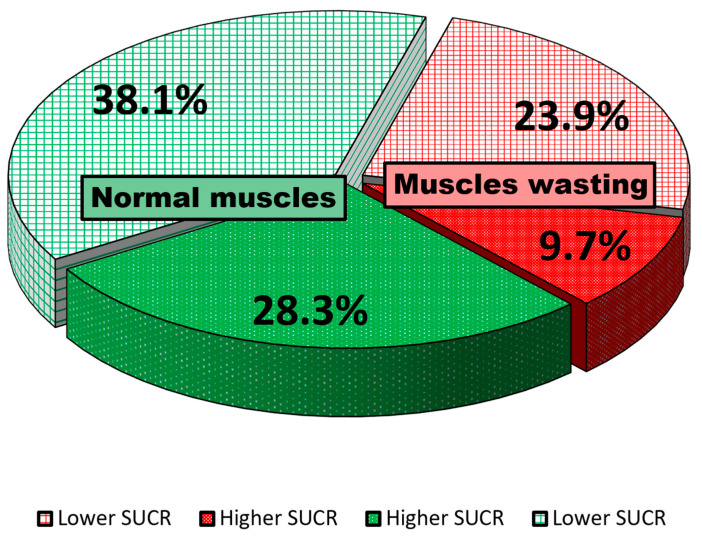
The percentages of patients with distinct skeletal muscle phenotypes as defined by ASMI and SUCR values.

Among the whole cohort, 242 (33.6%) patients had muscle-wasting features as defined by the most recent report of EWGOP. The remaining 478 (66.4%) had normal appendicular muscle mass. One of the muscle-wasting phenotypes was defined as coexisting with SUCR levels < 1.314 g/L, and was found in 172 patients (26.9%) out of the whole cohort. The alternative muscle-wasting phenotype with SUCR levels equal or above 1.314 g/L was recognized in 70 patients (9.7%). In patients with normal appendicular muscle mass, the phenotype with lower SUCR levels was present in 274 (38.1%) patients, while in the remaining 204 (28.3%) of patients, the phenotype of normal skeletal muscle mass met higher levels of SUCR (Figure 1).

### 3.3. Differences between Clinical and Laboratory Characteristics of Study Groups (Phenotypes)

The comparison of patients according to skeletal muscle phenotypes was shown in Table 2. There were no differences in the distribution of age, gender and HF etiology (*p* > 0.05). 

We identified some differences across study groups. There were differences in the duration of HF and HF symptoms. In patients with muscle-wasting phenotypes, the symptoms were more advanced, as shown by lower blood pressure, higher NYHA class, more compromised left ventricle ejection fraction and lower peak oxygen consumption on symptom-limited treadmill exercise (Table 2). 

Anthropometric and body composition characteristics were different in study groups. Before HF onset, patients with muscle-wasting phenotypes had smaller BMI, more of these patients had low baseline BMI and they lost more weight during HF. As a result, their index BMI was also lower (*p* < 0.001). Furthermore, the proportion of patients with degree of weight loss (>20%), strongly suggesting malnutrition was bigger in patients with muscle-wasting phenotypes. The trajectory of weight change, as reflected by catabolic/anabolic balance, showed a shift toward more catabolic profile in patients with muscle-wasting phenotypes. Additionally, catabolic component of weight trajectory was higher in muscle-wasting groups, while the anabolic component one only trended toward smaller values in these groups. Except weight loss, all were lower in patients classified with muscle-wasting phenotypes. 

Laboratory profile suggested more advanced stage of HF among patients with low ASMI. Serum levels of NTproBNP were higher in these patients, while sodium and eGFR were lower. Additionally, patients with muscle-wasting phenotypes had higher levels of cystatin C, but the ratio of creatinine-to-cystatin-C was lower. Moreover, GGTP serum activity reflecting liver injury showed higher values in patients with muscle-wasting phenotypes. Despite similar hemoglobin levels in all muscle phenotypes, some alterations in red cells indices were found. In patients with muscle wasting all indices, except mean corpuscular volume of erythrocytes, were lower than in patients with preserved muscles (Table 2). 

The prevalence of comorbidities among muscle wasting or preserved muscles phenotypes did not vary. However, we have noticed some differences in therapy. Patients with muscle wasting received either less frequently ACEI/ARB/ARNI therapy, or the dosages of these drugs were lower. Furthermore, despite number of patients were similar among study groups, the dosages of beta-blockers were lower. The same was true with regard to loop diuretics. The dosage of loop diuretics was higher in patients with muscle wasting (Table 2). 

### 3.4. Post Hoc Comparisons 

We have identified only minor differences between muscle-wasting phenotypes. In comparison to the phenotype with higher SUCR, the phenotype with lower SUCR lost more weight during HF and their catabolic to anabolic balance was shifted toward catabolism (*p* < 0.001). Additionally, these patients had worse liver test and lower MCH and were administered lower dose of ACEI/ARB/ARNI (Table 2). Among 33.6% patients having low ASMI, 26% had BMI > 25 kg/m^2^.

In patients with preserved muscles, those with lower SUCR as compared to higher SUCR had worse NYHA class, more weight loss and higher catabolic component of weight trajectory and the metabolic balance shifted more toward catabolism dominance, lower estimated GFR, higher cystatin C and lower-creatinine-to-cystatin-C ratio (Table 2).

### 3.5. The Estimated Outcome in Study Phenotypes 

The MAGGIC estimation of 3-year survival in predefined phenotypes showed higher mortality risk in phenotypes with muscle wasting as compared to phenotype with preserved muscles and higher SUCR (Group 4). However, the post hoc analysis disclosed significantly higher risk in the muscle-wasting phenotype coexisting with lower SUCR as compared to muscle wasting with higher SUCR. There were no statistical differences in estimated mortality risk between phenotypes of preserved muscles defined according to various levels of SUCR. 

### 3.6. The Observed Mortality 

**Figure 2:**
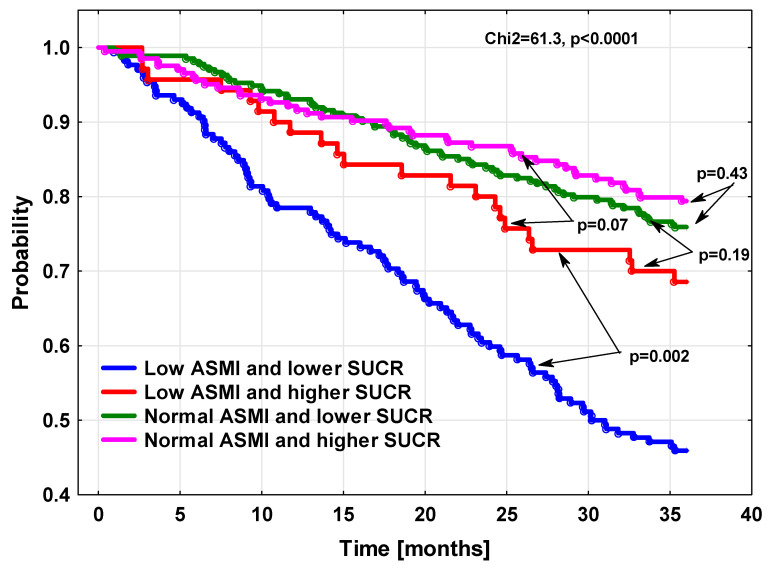
The cumulative probability of survival in HF patients according to appendicular skeletal mass index and SUCR value.

**Figure 3:**
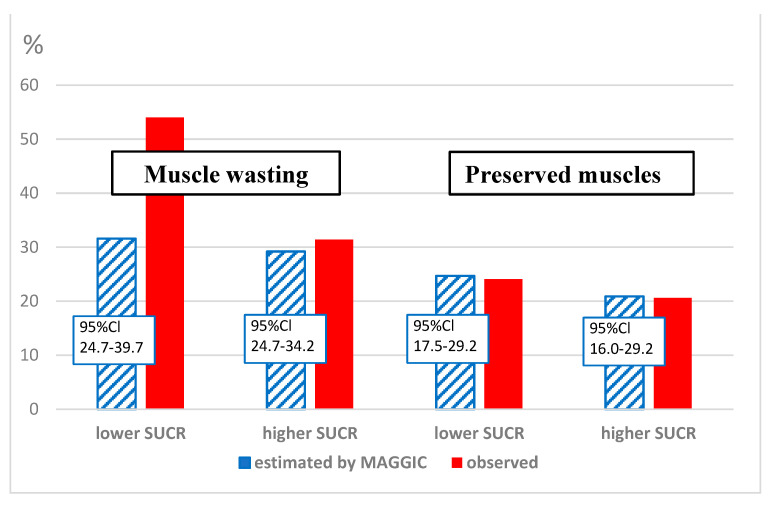
The comparison of estimated 3-year risk of death and true mortality rates in patients with different muscle phenotypes.

In both phenotypes with preserved muscles but also in the muscle-wasting phenotype with higher SUCR, the mortality was not statistically different, as demonstrated by Kaplan–Meier survival curves (Figure 2), or based on direct comparisons of death rates in study groups. In contrast to these three phenotypes, the risk of death for the muscle-wasting phenotype coexisting with lower SUCR was much higher. There was also significant difference between mortality risk in this particular phenotype, as estimated based on the MAGGIC algorithm and true death rates (Figure 3).

### 3.7. The Crude and Adjusted Mortality Risk Models

In unadjusted Cox analysis, taking as a reference the phenotype with worse outcome, that is muscle wasting with lower SUCR (Group 1), the risk in all other phenotypes was significantly reduced by approximately 50–70% (Table 3). The risk difference between this and other phenotypes was slightly lower but still significant when we adjusted the model by MAGGIC score. In our final model, the risk was estimated after additional adjustment for weight loss, anabolic and catabolic components of weight trajectory as well as creatinine-to-cystatin-C ratio. Even in this last model, the risk of death in the three aforementioned phenotypes was significantly reduced by about 40% as compared to the muscle-wasting phenotype with lower SUCR (Table 3). 

## 4. Discussion

The main findings of our study are fourfold. First, we have showed prognostically different muscle-wasting phenotypes according to SUCR, which is simple to measure and easily available in everyday practice. Second, we have demonstrated that MAGGIC, a commonly used HF prognostic score, failed to capture extremely elevated mortality risk in up one-fourth of HF patients having the aforementioned, newly identified muscle-wasting phenotype. Third, we have identified the muscle-wasting phenotype with relatively good prognosis, but it was hardly identified based on clinical and laboratory variables. The main laboratory feature of this phenotype is a higher level of SUCR. Finally, we have found that in patients with preserved SM, lower SUCR does not carry significant prognostic information. The mentioned findings are of great clinical relevance, as they can allow clinicians to better adjust therapy to patients’ true mortality risk, potentially leading to more favorable prognosis. 

According to data currently available, muscle wasting in HF may be present in up to 34% of patients and is associated with much higher morbidity and mortality [25,26,27]. In some patients, muscle wasting may be masked by normal or even elevated BMI, and weight loss may be marginal. In fact, among 33.6% patients having low ASMI in our relatively big study, more than a quarter had BMI > 25 kg/m^2^. Additionally, these patients, as many as 77% had weight loss in HF < 5%. These intriguing facts highlight the importance of body composition studies in HF patients. 

Although in the general population, muscle mass and strength are significantly correlated, only 13.3% of the variation in muscle strength can be explained by muscle mass after adjustment for age and gender [28]. In patients with HF, this relationship is even worse, as the association between mass and strength is much weaker [28]. The unit increase of muscle mass in HF is associated with 20% lower increase in muscle strength in comparison to healthy adults [28]. All these observations point to other reasons for the dissociation between mass and function in HF. 

Although low ASMI is doubtlessly associated with worse clinical outcome, muscle quality and function as represented by strength, gait speed or other measures more closely predict the risk in general population [29]. In HF, low strength and worse function may coexist with normal muscle mass [30]. That is why guidelines for patients at risk of muscle wasting recommend muscle strength testing as a first step in clinical workup [23,31]. However, these measurements require certified dynamometers and trained staff to obtain valid results. Additionally, a recent study showed that indexes integrating both muscle function and body composition may better predict slower walking speed, the parameter closely related to lower quality of life and higher morbidity and mortality [32]. 

Creatine is an essential molecule to maintain constant ATP provision to working muscles through the phosphocreatine-shuttling mechanism. However, it is not synthetized in SM and the heart. About 50% of creatine comes from diet, while the remaining part is synthetized in a two-step reaction; initially in the kidneys and pancreas and later in the liver. First, L-arginine and glycine is required for enzymatic synthesis of guanidinoacetic acid (GAA). Then, GAA is transported by blood to the liver, where methionine serves for methylation of GAA to creatine. All creatine necessary for proper muscle function must by transported in the blood, after which it is taken up by myocytes. Independently of available creatine pool size, about 2% is passively dehydrated into creatinine and excreted in urine daily [18]. 

In HF patients, especially during exercise [33], dietary provision [34], gut absorption [35] and effective transport of creatine to hypoperfused muscles might be impaired. Additionally, availability of creatine may be hampered by low serum levels of L-arginine, glycine and methionine [36], as well as poor metabolic function of kidneys and the liver; all these abnormalities are frequently encountered in HF [37]. Thus, decreased creatine pool, already shown in HF [38], may be responsible for both reduced output of creatinine in urine and energetic constrains to the heart and SM, finally resulting in worse prognosis. Therefore, one may argue that urinary creatinine might serve as a useful biomarker of muscle mass but also their function. The SUCR level may be one such biomarker candidate.

Only a few studies analyzed SUCR levels in patients with HF [15,16,17]. All of them have found positive association between lower SUCR and higher mortality, but only in one study, direct measurements of muscle mass [17] as a potential confounder was included in risk analysis. In this study, fat-free mass only weakly correlated with SUCR. No data have been published so far concerning the association between muscle strength and SUCR. 

In one of the studies mentioned, there was a negative correlation between weight loss and SUCR. In our study, such a correlation also existed, but it was the strongest in the phenotype with preserved muscles and low SUCR This finding is difficult to explain. We suppose that the SUCR level in this phenotype better reflects the functional than the structural aspect of myopathy in HF. This possibility is underscored by higher cystatin C and a lower creatinine/cystatin C ratio, known to correlate more closely with functional impairment than with the presence of muscle wasting in dialyzed patients [39]. Such an explanation is further supported by a worse NYHA class in this particular phenotype as compared to patients with preserved muscles and higher SUCR. 

The lower spot urinary creatinine muscle-wasting phenotype was associated with a particularly poor outcome, and this excessive risk was not captured by the MAGGIC score. The clinical and laboratory profile of these patients differed little from the phenotype with low ASMI but higher SUCR. Of note is the higher activity of GGTP, meaning more liver injury and lower MCH levels, suggesting iron depletion. Although hemoglobin was similar in all phenotypes, we cannot exclude the possible iron depletion as an underlying cause of worse functional performance, as proven by the NYHA class, lower blood pressure and more catabolic profile. All these characteristics are more prevalent in patients with iron depletion [40]. Of particular interest is the association between iron depletion and the lower ratio of phosphocreatine to ATP within the heart, which has been recently documented in HF patients [41]. They did not measure urinary creatinine, but one may speculate that lower creatine metabolism might have reduced creatinine production, and thus, lower creatinine excretion in urine. If confirmed, the finding could provide an additional argument for low SUCR as an index of both low muscle mass and more global energetic impairment. 

The patients in wasting with lower SUCR were treated with a lower dose of ACEI/ARB/ARNI. In the fully adjusted model, three-year mortality risk in people with low ASMI and low SUCR phenotype was at least 40% higher compared to all other phenotypes. This difference cannot be explained by lower dosing of these drugs, because a much higher difference in treatment intensity that was reported recently in a large Swedish registry was associated with only a 25a% reduction of all-cause mortality risk [42]. 

The patients with a preserved SM mass and lower SUCR had a statistically similar risk compared to patients with higher SUCR. This prognostic similarity occurred despite a worse NYHA class and more catabolic profile of these patients. The true and estimated MAGGIC values in both phenotypes were also identical. This intriguing observation may highlight importance of preserved energetic metabolism, as shown by higher SUCR in HF. The more precise explanation of underlying mechanism remains to be established. 

## 5. Study Limitations

Our study has several limitations. The cross-sectional study design does not allow analysis of causality. The use of DEXA for measuring fat-free mass in patients with HF might have been a source of measurement imprecision. Despite our best efforts to establish edema-free status, water excess in some patients could not be ruled out. If present, it may have distorted body composition analysis because DEXA recorded extracellular water as fat-free mass. DEXA scanning cannot recognize fat tissue infiltration within skeletal muscles, which may reduce metabolic activity despite preserved muscle mass. Fat infiltration might have a negative effect on muscle function which was not measured in our study. No evaluation of muscle function is also a limitation. The data on diet potentially affecting creatine provision, as well as more precise information on gut absorptive function, were also not available. The urine samples were obtained only once on the index day, which may constitute a significant bias. The specific urine gravidity might have distorted SUCR measurement independently of creatine metabolic status. We were not able to take this effect into account. 

To sum up, the measurement of SUCR in HF can identify clinical phenotypes with SM wasting, but with a strikingly different risk of death that is actually not captured by the MAGGIC score. The higher level of SUCR was associated with a similar risk independently of the presence of muscle wasting. As the analysis of SUCR is easy to perform, available everywhere and cheap, it should be further tested as a potentially useful biomarker which may precisely phenotype HFrEF patients independently of their SM status. 

## Figures and Tables

**Table 1:** Clinical and laboratory characteristics of study group and comparison of patients according to presence or absence of muscle wasting.

**Part 1**	
Feature	All patients N = 720
Baseline demographics and functional tests	
Age (years)	52.3 ± 10
Gender—% males (%)	86
HF etiology—ischemic (%)	57.1
NYHA class I/II/III/IV (%)	5/35/51/9
Duration of HF (months)	35; (13–71)
Systolic BP (mmHg)	107 ± 16
MVO2 (mL/kg min)	14.2; (11.6–17.3)
LVEF (%)	24.3 ± 7.3
Anthropometrics and body composition	
Pre-HF BMI (kg/m^2^)	28.3 ± 4.7
% pre-HF BMI < 20 or <22 kg/m^2^ if < or ≥70 years (%)	2.1
IndexBMI (kg/m^2^)	26.2 ± 4.5
Weight loss from pre-HF BMI until index BMI (%)	7.9; (1.1–14.3)
Weight loss > 20 (%)	10.1
Catabolic component of weight trajectory (%)	−11.5; (−18.2–−5.3)
Anabolic component of weight trajectory (%)	3.6; (0.0–9.3)
Catabolic/anabolic balance (%)	−16.3; (−24.7–−10.1)
Fat mass (kg/m^2^)	7.2; (5.6–9.0)
Fat mass (%)	27.5 ± 7.8
Fat-free mass (kg/m^2^)	17.7; (16.0–19.4)
ASMI (kg/m^2^)	7.4 ± 1.2
**Part 2**	
Laboratory tests	
Hemoglobin (mmol/L)	8.7 ± 1.1
MCV (fL)	91.2; (87.7–94.9)
MCH (pg/cell)	30.8; (29.6–32.0)
MCHC (g/dL)	20.9 (20.4–21.5)
NTproBNP (pg/mL)	1470; (679–3287)
Creatinine (mg/dL)	0.98; (0.83–1.22)
eGFR_MDRD_ (mL/min*1.73m^2^)	81; (66–97)
GGTP (iu)	53.5 (28–109)
Cystatin C (mg/L)	0.94; (0.79–1.15)
Creatinine/cystatin ratio (number)	1.04; (0.89–1.23)
Sodium (mmol/L)	136; (134–138)
hCRP (mg/dL)	2.8; (1.2–6.7)
Spot urinary creatinine (g/L)	1.04; (0.55–1.59)
Comorbidities	
Hypertension (%)	55
Diabetes mellitus type 2 (%)	29
Hypercholesterolemia (%)	61
Hypertriglycerydemia (%)	43
History of smoking (%)	74
Therapy	
ACEI/ARB/ARNI (% treated/% of recommended dose)	94/50; (25–100)
BB (% treated/% of recommended dose)	98/50; (33–67)
MRA (% treated/% recommended dose)	95/50; (50–50)
Loop diuretics (% treated/mg of furosemide eq.)	91/80; (40–120)
Mortality prediction and observed outcome	
MAGGIC mortality prediction at 3y (mean ± 95% CI; %)	24.7; (19.1–34.2)
True mortality at 3 years (%)	31.0
Estimated/observed ratio	0.88

Legend: MCV—Mean corpuscular volume, MCH—Mean corpuscular hemoglobin, MCHC—Mean corpuscular hemoglobin concentration, NTproBNP—N-terminal propeptide of brain-type natriuretic peptide, eGFR_MDRD_—estimated glomerular filtration rate based on Modification of Diet in Renal Disease equation, GGTP—gamma glutamyl transpeptidase, hsCRP—high-sensitivity C-reactive protein, ACEI/ARB/ARNI—angiotensin converting enzyme inhibitor/angiotensin receptor blocker/angiotensin receptor neprylisine inhibitor, BB—beta-blocker, MRA—mineralocorticoid receptor antagonist, Eq.—equivalent, MAGGIC—Meta-analysis Global Group in Chronic Heart Failure, CI—±95% confidence intervals.

**Table 2:** Clinical and laboratory characteristics and comparison of patients divided according to ASMI and SUCR.

	Study Groups				
Feature	Low ASMI—Muscle-Wasting Phenotype		Normal ASMI—Preserved Muscles Phenotype		*p*-value
	Group 1 (N = 172) Lower SUCR	Group 2 (N = 70) Higher SUCR	Group 3 (N = 274) Lower SUCR	Group 4 (N = 204) Higher SUCR	
Baseline demographics and functional tests					
Age (years)	52.9 ± 12	51.5 ± 12	52.2 ± 10	52.3 ± 9	0.801
Gender—% males (%)	84.9	90.0	83.2	89.2	0.209
HF etiology—ICM (%)	55.2	60.0	59.1	54.9	0.718
NYHA class I/II/III/IV (%)	2/16/62/20 @	1/27/61/10 @ A	6/38/49/8 Y	6/50/42/2	<0.001
Duration of HF (months)	40; (13–84)	34; (11–68)	32; (13–67) &	38; (12–67)	0.043
Systolic BP (mmHg)	101 ± 14.8 @	103 ± 14.2 @	109 ± 15.7	111 ± 15.7	<0.001
MVO_2_ (ml/kg min)	13.3; (11.0–16.5) @	12.8; (10.1–15.5) #	15.1; (12.1–18.4)	14.5; (11.9–17.8)	<0.001
LVEF (%)	22 ± 8 #	22 ± 7 &	25 ± 7	25 ± 7	0.001
Anthropometrics and body composition					
Pre-HF BMI (kg/m^2^)	25.7 ± 3.9 @	26.3 ± 3.9 @	29.8 ± 4.6	29.3 ± 4.7	<0.001
% pre-HF BMI <20 kg/m^2^ if < 70 years or <22 kg/m^2^ if ≥ 70 years (%)	5.2 @	5.7 @	0.7	0.0	<0.001
IndexBMI (kg/m^2^)	22.6 ± 3.4 @	23.8 ± 3.1 @C	27.7 ± 4.0	28.0 ± 4.1	<0.001
Weight loss from pre-HF BMI until index BMI (%)	11.4; (5.1–17.5) @	8.9; (3.1–15.3) &A	7.3; (0.1–13.5) X	4.7; (−1.2–11.3)	<0.001
Weight loss >20 (%)	18,6 @	10.0 #B	8.4 X	5.4	<0.001
Catabolic component of weight trajectory (%)	−15.1; (−20.9–−8.7) @	−12.4; (−18.9–−9.1) @	−11.1; (−18.2–−4.8) X	−8.5; (−15.1–−3.9)	<0.001
Anabolic component of weight trajectory (%)	2.5; (0.0–7.7) &	2.8; (0.0–9.4)	4.2; (0.0–10.0)	4.8; (0.0–9.9)	0.095
Catabolic/anabolic balance (%)	−18.1; (−25.8–−11.5) @	−18.3; (−27.1–−11.1) @A	−15.7; (−23.3–10.0) X	−14.7; (−22.1–−8.8)	0.006
Fat mass (kg/m^2^)	6.0; (4.2–7.8) @	6.6; (5.0–8.1) @	7.7; (6.3–9.2)	7.7; (6.1–9.9)	<0.001
Fat mass (%)	26.2 ± 9.3	27.2 ± 9.1	28.2 ± 6.7	27.8 ± 7.2	0.059
Fat-free mass (kg/m^2^)	15.8; (14.0–16.8) @	15.9; (14.9–17.0) @	18.7; (17.5–20.0)	18.5; (17.6–20.1)	<0.001
ASMI (kg/m^2^)	6.3 ± 0.8 @	6.4 ± 0.7 @	7.9 ± 1.0	8.1 ± 1.0	<0.001
Laboratory tests					
Hemoglobin (mmol/L)	8.7 ± 1.2	8.7 ± 1.1	8.7 ± 1.0	8.7 ± 1.0	0.971
MCV (fL)	90.4; (87.1–94.6)	92.2; (87.9–95.9)	91.5; (87.5–95.3)	91.1; (88.5–94.7)	0.309
MCH (pg/cell)	30.4; (29.1–31.8) @	31.0; (30.2–32.6) B	30.8; (29.6–32.2)	30.9; (30.1–32.1)	0.022
MCHC (g/dL)	20.8; (20.2–21.3) &	21.0; (20.5–21.7)	20.9; (20.4–21.9)	21.1; (20.5–21.5)	0.064
NTproBNP (pg/mL)	2744;(1426–4938 @	1989;(989–4115) @	1145; (639–2547)	1093; (536–2169)	<0.001
Creatinine (mg/dL)	1.02; (0.83–1.28)	0.99; (0.85–1.15)	0.94; (0.82–1.22)	0.98; (0.85–1.18)	0.700
eGFR_MDRD_ (mL/min*1.73 m^2^)	75; (56–90) @	79; (62–94) #	82; (67–96) Z	87; (74–101)	<0.001
GGTP (iu)	93 (39–177) @	65 (31–119) B	44 (27–91)	42 (24–79)	<0.001
Cystatin C (mg/L)	1.05; (0.85–1.34) @	0.97; (0.82–1.21) @	0.93; (0.8–1.13) Z	0.88; (0.76–1.03)	<0.001
Creatinine/cystatin ratio (number)	0.95; (0.82–1.15) @	1.04; (0.86–1.18) @	1.02; (0.88–1.21) Z	1.14; (0.99–1.31)	<0.001
Sodium (mmol/L)	135; (131–137) @	136; (133–138) &	137; (135–138)	137; (135–139)	<0.001
hCRP (mg/dL)	3.5; (1.3–9.3)	3.5; (1.4–8.3)	2.4; (1.1–5.3)	2.6; (1.2–6.5)	0.357
Spot urinary creatinine (g/L)	0.68; (0.37–0.98) @	1.68; (1.42–2.24)	0.68;(0.36–0.99) Z	1.73; (1.53–2.15)	<0.001
Comorbidities					
Hypertension (%)	52	43	58	56	0.130
Diabetes mellitus type 2 (%)	31	26	32	25	0.337
Hypercholesterolemia (%)	54	53	64	64	0.066
Hypertriglycerydemia (%)	36	39	48	43	0.088
History of smoking (%)	76	67	70	79	0.163
Therapy					
ACEI/ARB/ARNI (% treated)	88 @	93	98	95	<0.001
ACEI/ARB/ARNI (% of target dose)	38; (12.5–50) @	50; (25–100) A	50; (25–100)	50; (25–100)	<0.001
BB (% treated)	95	99	99	98	0.070
BB (% target)	33; (25–67) @	50; (33–67)	50; (33–67)	58; (33–67)	<0.001
MRA (% treated)	94	96	96	94	0.530
MRA (% of target dose)	50; (50–100)	50; (50–100)	50; (50–50)	50; (50–50)	0.056
Loop diuretics (% treated)	91	96	90	89	0.406
Loop diuretics (mg of furosemide eq.)	120; (80–160) @	120; (40–160) #	80; (40–120)	80; (40–120)	<0.001
Mortality prediction and observed outcome					
MAGGIC mortality prediction at 3y * (%)	31.6; (24.7–39.7) @	29.2; (24.7–34.2) @	24.7; (17.5–29.2)	20.9; (16.0–29.2)	<0.001
True mortality at 3 years (%)	54.0 @ C	31.4 B	24.1	20.6	<0.001
Estimated/observed ratio	0.66	1.08	0.91	1.07	

Legend: MCV—Mean corpuscular volume, MCH—Mean corpuscular hemoglobin, MCHC—Mean corpuscular hemoglobin concentration, NTproBNP—N-terminal propeptide of brain-type natriuretic peptide, eGFR_MDRD_—estimated glomerular filtration rate based on Modification of Diet in Renal Disease equation, GGTP—gamma glutamyl transpeptidase, hsCRP—high-sensitivity C-reactive protein, ACEI/ARB/ARNI—angiotensin converting enzyme inhibitor/angiotensin receptor blocker/angiotensin receptor neprylisine inhibitor, BB—beta-blocker, MRA—mineralocorticoid receptor antagonist, Eq.—equivalent, MAGGIC—Meta-analysis Global Group in Chronic Heart Failure, *—mean ±95% confidence intervals. Multiply comparisons: & *p* ≤ 0.05, Normal ASMI and higher SUCR as reference, A *p* ≤ 0.05, comparison of Low ASMI and higher SUCR vs. Low ASMI and lower SUCR, X *p* ≤ 0.05, comparison normal ASMI and lower SUCR vs. normal ASMI and higher SUCR, # *p* ≤ 0.01, Normal ASMI and higher SUCR as reference, B *p* ≤ 0.01, comparison of Low ASMI and higher SUCR vs. Low ASMI and lower SUCR, Y *p* ≤ 0.01, comparison normal ASMI and lower SUCR vs. normal ASMI and higher SUCR, @ *p* < 0.001, Normal ASMI and higher SUCR as reference, C *p* < 0.001, comparison of Low ASMI and higher SUCR vs. Low ASMI and lower SUCR, Z *p* < 0.001, comparison normal ASMI and lower SUCR vs. normal ASMI and higher SUCR.

**Table 3:** The hazard ratio for all-cause mortality in study groups compared to a group with normal ASMI and higher SUCR.

	Three-Year Mortality Risk Cox Regression Analysis			
Feature	Low ASMI and Lower SUCR (Ref.)	Low ASMI and Higher SUCR	Normal ASMI and Lower SUCR	Normal ASMI and Higher SUCR
	Hazard Ratio ± 95% CI, *p*-Value			
Raw model	1.0	0.48; (0.30–0.77), *p* = 0.002	0.34; (0.26–0.48), *p* < 0.0001	0.30; (0.21–0.43), *p* < 0.0001
Adjusted for three-year MAGGIC risk	1.0	0.57; (0.36–0.92), *p* = 0.02	0.52; (0.37–0.73), *p* = 0.0002	0.46; (0.30–0.68), *p* < 0.0001
Adjusted as above + weight loss, catabolic and anabolic components, creatinine-to-cystatin-C ratio	1.0	0.59; (0.37–0.94), *p* = 0.03	0.55; (0.39–0.78), *p* = 0.0007	0.55; (0.37–0.84), *p* = 0.005

## Data Availability

The data presented in this study are available on request from the corresponding author.
